# Special Issue “HPV in the Head and Neck Region”

**DOI:** 10.3390/v13122452

**Published:** 2021-12-06

**Authors:** Tina Dalianis, Christian von Buchwald, Anders Näsman

**Affiliations:** 1 Department of Oncology-Pathology, Karolinska Institutet, Bioclinicum J6:20, Karolinska University Hospital, 171 64 Stockholm, Sweden; 2 Department of Otorhinolaryngology—Head and Neck Surgery and Audiology, Copenhagen University Hospital, Rigshospitalet, 2100 Copenhagen, Denmark; Christian.von.Buchwald@regionh.dk; 3 Department of Clinical Pathology, CCK R8:02, Karolinska University Hospital, 171 64 Stockholm, Sweden

Previously, human papillomaviruses were best known for causing diseases in the genital tract, where high-risk types may cause, e.g., cancer of the cervix uteri, while low risk types could cause condylomas. Recently, more efforts have also been made to disclose their effects in the head and neck region. This Special Issue on “HPV in the head and neck region” includes, one editorial, eight reviews, one brief report and two systematic reviews that contribute to the overall knowledge of HPV in the head and neck region [1,2,3,4,5,6,7,8,9,10,11,12].

The editorial describes focal epithelial hyperplasia (FEH) or Heck’s, which is a rare benign condition, primarily associated with HPV13 or 32 or both, usually presented as multiple asymptomatic whiteish to mucosal-colored, soft, papular or nodular lesions in the oral cavity, needing treatment if inconveniently growing [1]. Findings in relation to HPV genotypes, geographical distribution, comorbidities and treatment, and recent case reports within the field are described, including an FEH lesion infected with the low-risk HPV90.

One review covers the issue of HPV vaccination of boys and young men, a vaccine previously mainly considered for females, but, as indicated here, an issue important also for males [2]. The hurdles and attitudes for and against HPV vaccination of boys and men are portrayed and the authors conclude that boys should be included in national immunization programs and be offered catch-up vaccination.

A second review covers HPV-associated benign squamous cell papillomas (SCP) in the upper aero-digestive tract, their malignant potential and the varying association to HPV infection depending on sites [3]. A review on HPV-related neoplasia of the ocular adnexa is also included, indicating that the role of HPV in squamous cell tumors arising in the lacrimal drainage system and the eyelid is still uncertain and further studies are needed [4].

A separate review and systematic literature search dealt with the effects of HPV vaccines on oral and oropharyngeal HPV infections and reported a decrease in HPV vaccine-type infections in HPV vaccinated study participants [5].

Another review topic was HPV and squamous cell carcinoma of unknown primary in the head and neck region, and its clinical implications, concluding that the detection of HPV in cytological specimens was indicative of a primary oropharyngeal squamous cell carcinoma (OPSCC) [6]. Advances in diagnostics, e.g., transoral robotic surgery and transoral laser microsurgery, have increased the successful identification of the primary tumor site, thereby harboring a potential for improved treatment options [6].

Striving towards improved treatment options of OPSCC in the era of check point inhibitors was also covered and this review suggests that indeed new options for the future will fuel the next evolution of treatment for OPSCC [7]. Additionally, regarding treatment, a review is included on the relevance of HPV in head and neck cancer and what remains in 2021 from a clinician’s point of view [8]. Here, the correct diagnosis of an HPV positive tumor, with inclusion not only of p16^INK4A^ but also HPV DNA positivity is emphasized. In addition, some drawbacks of the 8th TNM-classification of Malignant tumors system are discussed, and the importance of prophylactic HPV vaccination is underlined. Along a similar line with regard to improved treatment is a review on the attempts to identify additional prognostic and driver genes in HPV positive tonsillar and base of tongue cancer in order to better personalize patient treatment [9].

A brief report, with a systematic review on HPV-related multiphenotypic sinonsasal carcinoma (HMSC), a new emerging tumor entity according to the World Health Organization (WHO) was also presented [10]. The authors indicate that this disease may possibly occur also outside the sinonasal region; however, they also suggest that HMSC needs to be better characterized before it can be justified as its own tumor entity.

A systematic review on the accuracy of HPV detection in OPSCC was also included [11]. An overall high accuracy for HPV detection in tumor tissue was found, irrespective of the method, and HPV detection in blood was also indicated as promising for HPV detection.

Finally, a systematic review on the HPV-positivity in OPSCC worldwide presented variations in HPV occurrences with tendencies for the highest occurrences in Northern Europe, the USA, Lebanon, China, and South Korea [12].

With this, we would like to thank all authors for their valuable contributions to this Special Issue. It has been extremely interesting and rewarding to learn more of this relatively new field. Together, we hope we will continue our endeavors to increase the knowledge in the field in order to both better prevent disease and individualize patient treatment.

## Data Availability

Not applicable.
